# What Is Next for Refractory Colorectal Cancer CRC? Looking Beyond SUNLIGHT, FRESCO2, RECURSE and CORRECT

**DOI:** 10.3390/ijms26062522

**Published:** 2025-03-11

**Authors:** Sara Cherri, Michela Libertini, Silvia Noventa, Ester Oneda, Fausto Meriggi, Alberto Zaniboni

**Affiliations:** Department of Clinical Oncology, Fondazione Poliambulanza, 25124 Brescia, Italy; michela.libertini@poliambulanza.it (M.L.); silvia.noventa@poliambulanza.it (S.N.); ester.oneda@poliambulanza.it (E.O.); fausto.meriggi@poliambulanza.it (F.M.); alberto.zaniboni@poliambulanza.it (A.Z.)

**Keywords:** colon cancer, chemotherapy, refractory, immunotherapy, target therapy, mitomycin C, rechallenge

## Abstract

The treatment landscape of metastatic colorectal cancer (mCRC) has undergone significant evolution, with the introduction of targeted therapies and immunotherapy dramatically altering the management of microsatellite instability-high (MSI-H) tumors. However, the majority of patients, particularly those with microsatellite-stable (MSS) disease, remain refractory to immunotherapy, necessitating the exploration of alternative therapeutic strategies. This review summarizes the current treatment options for heavily pretreated mCRC patients who are not eligible for targeted therapies or clinical trials. Approved therapies for refractory mCRC, including regorafenib, trifluridine/tipiracil (FTD/TPI), and fruquintinib, demonstrate modest survival benefits but are often associated with significant toxicities. Additionally, innovative approaches targeting specific mutations such as KRAS G12C, HER2 amplification, and BRAF V600E are discussed, highlighting emerging combination regimens with immune checkpoint inhibitors and other agents to overcome resistance mechanisms. The potential of rechallenge strategies using previously administered therapies, such as oxaliplatin and anti-EGFR agents, is examined, supported by retrospective and prospective studies. Furthermore, the role of older drugs like mitomycin C in combination with capecitabine is revisited, offering insights into their viability in advanced treatment settings. Ongoing clinical trials with novel agents and combinations are expected to provide further clarity on optimizing sequential treatment regimens and personalizing therapy for mCRC patients. This review emphasizes the need for comprehensive molecular profiling and shared decision-making to improve outcomes and quality of life in this challenging patient population.

## 1. Introduction

In recent years, the treatment of solid tumors has witnessed the emergence of numerous novel agents and targeted therapies across various disease settings and histotypes, reflecting the growing need for increasingly personalized treatment approaches. This evolution has rapidly transformed the management of many oncological conditions, affecting treatment protocols for both localized disease (through neoadjuvant and adjuvant strategies) and advanced stages. In metastatic disease, these advances have been integrated into treatment regimens and, in some cases, have gradually moved into first-line therapies, with the goal of achieving maximum benefit and early response—even within palliative care settings. In some instances, this has led to remarkable outcomes and durable complete responses, prompting a reevaluation of palliative treatment strategies for the future [1,2,3].

In metastatic colorectal cancer (mCRC), the treatment landscape is particularly complex, necessitating an intense effort to optimize available therapies in the metastatic setting—a process complicated by several factors. First, the subtype that has shown the most significant benefit in terms of practice-changing outcomes and complete responses is characterized by microsatellite instability (MSI), which permits the use of immune checkpoint inhibitors. Specifically, in these patients, pembrolizumab—administered both as first-line therapy and in subsequent lines—has dramatically altered the disease’s natural history. Notably, in the phase II KEYNOTE-164 trial, pretreated metastatic patients receiving pembrolizumab monotherapy achieved an objective response rate (ORR) of 33%. Moreover, the phase III randomized KEYNOTE-177 trial demonstrated that patients treated with pembrolizumab as first-line therapy experienced significantly longer progression-free survival (PFS) compared to those receiving chemotherapy (median, 16.5 months vs. 8.2 months; HR: 0.60; 95% CI: 0.45–0.80; *p* = 0.0002) [4,5]. However, only about 15% of CRC patients exhibit MSI-high status, limiting eligibility for these innovative treatments. Consequently, a significant portion of current research is dedicated to extending the benefits of immunotherapy to a larger group of mCRC patients, particularly those with microsatellite-stable tumors—a challenge that remains unresolved.

Another complicating factor in mCRC treatment is the presence of multiple concurrent mutations, which can substantially reduce the effectiveness of targeted therapies by inducing both primary and secondary resistance. Numerous studies are underway to explore combinations of new agents to overcome this resistance and identify targeted treatments that can provide durable responses. Despite these vigorous research efforts, this issue remains unsolved.

A third aspect, which is the primary focus of this review, concerns the management of heavily pretreated patients in the metastatic setting who do not have access to targeted therapies—either due to a lack of available options or the absence of clinical trials—but who nonetheless maintain a reasonable performance status. This review will examine recent studies on treatment strategies beyond the second line, including the use of antiangiogenic agents and various combination therapies, while also questioning the future role of standard chemotherapy and rechallenge strategies. The aim is to explore viable options for heavily pretreated patients who are currently excluded from ongoing innovative research yet represent a significant clinical challenge in daily oncology practice.

## 2. Refractory mCRC Disease

### 2.1. A Brief Summary of the Drugs Currently Indicated for Refractory Disease

In current clinical practice, the choice of the best treatment for patients with refractory colorectal cancer, i.e., pretreated in the first and second-line setting, is based on three fundamental assumptions: treatment lines already received, which depend on the disease’s characteristics, the clinical condition of the patient (especially their residual performance status), and lastly, but perhaps most importantly, the expected toxicity of the treatment (which thus impacts the patient’s quality of life). The choice of treatment cannot exclude these considerations, which should be discussed with the patient, as to date the best choice for third-line treatment is not known, nor is the optimal sequence of treatments for patients with refractory colon cancer. Certainly, patients with molecular characteristics susceptible to targeted therapy should be directed toward such treatments, but currently, it is rare that wild-type KRAS patients have not already undergone anti-EGFR therapy, and that patients with microsatellite instability (MSI) have not already received immunotherapy in earlier treatment settings. Rechallenge options are considered, and we will discuss this in the dedicated chapter. However, it must always be considered that pretreated patients may have poor bone marrow reserve, which makes them difficult candidates for chemotherapy rechallenge. Options for targeted therapies on other mutations (such as HER2, NTRK, RET) are extremely rare in current practice and are only available through clinical trials. Currently, the drugs indicated for patients with refractory mCRC are represented by two antiangiogenic drugs (regorafenib and fruquintinib) and an oral fluoropyrimidine (trifluridine/tipiracil), administered either as monotherapy or in combination with bevacizumab (see Table 1).

#### 2.1.1. Correct Trial and Regorafenib

Regorafenib is a multikinase inhibitor of tyrosine kinases involved in tumor angiogenesis mechanisms (e.g., PDGFR, FGFRs 1–2, VEGFRs 1–3, TIE2), tumor proliferation (e.g., RET, RAF, KIT), tumor microenvironment, and metastasis processes (VEGFR2–3, PDGFR), approved by the FDA and EMA for the treatment of refractory mCRC. The multicenter, randomized, placebo-controlled phase 3 CORRECT study showed that patients with refractory mCRC treated with regorafenib had a significant improvement in overall survival (OS), progression-free survival (PFS), and objective response rate (ORR) [6]. Patients were randomized to receive 160 mg of regorafenib or placebo once daily, along with best supportive care (BSC), on days 1 to 21 of a 28-day cycle. The study found that OS was more than 1.4 months longer for patients in the regorafenib + BSC group compared to those in the placebo + BSC group (HR 0.77; 95% CI, 0.64–0.94; *p* = 0.0052), and PFS was more than 0.2 months longer for the regorafenib + BSC group (HR 0.49; 95% CI, 0.42–0.58; *p* < 0.0001). These data were confirmed in an Asian population in the CONCUR study [7]. Although regorafenib proved to be an interesting molecule, it exhibited significant toxicity, with 93% of patients in the regorafenib arm experiencing an adverse event of any grade. Common side effects included fatigue, hand-foot syndrome (HFS), and hypertension. This led to the search for dose optimization strategies, including the multicenter, open-label phase 2 ReDOS study, where patients were randomly assigned to receive 80 mg of regorafenib daily, with weekly dose escalation, or the standard dose of 160 mg daily for 21 consecutive days in a 28-day cycle. The incidence of grade 3 adverse events commonly associated with regorafenib, particularly fatigue, HFSR, and hypertension, was numerically lower in the dose-escalation group compared to the standard-dose group. Today, international guidelines recommend using the dose-escalation strategy, as suggested by the ReDOS study, as an alternative dosing approach [8].

#### 2.1.2. RECOURSE, SUNLIGHT and Trifluridine/Tipiracil (FTD/TPI)

Trifluridine/tipiracil (FTD/TPI), an oral chemotherapeutic agent, specifically a third-generation fluoropyrimidine, is approved by the FDA and EMA for the treatment of refractory mCRC. The RECOURSE study is a multicenter, randomized, double-blind, placebo-controlled trial that compared the FTD/TPI + BSC arm with the placebo + BSC arm [9]. The FTD/TPI regimen involves oral administration twice a day on days 1 to 5 and 8 to 12 of a 28-day treatment cycle. The treatment with FTD/TPI demonstrated a benefit in both OS and PFS compared to placebo. Specifically, the median OS was 7.1 months for the FTD/TPI + BSC group compared to 5.3 months for the placebo + BSC group (HR, 0.68; 95% CI, 0.58–0.81; *p* < 0.001). Similar results were also published in a similar study conducted on an Asian population [10]. The rationale for combining the third-generation fluoropyrimidine with an anti-vascular agent led to the design of the SUNLIGHT study, which aimed to compare monochemotherapy with FTD/TPI to combination therapy with bevacizumab [11]. This study demonstrated the superiority of the combined therapy in terms of OS, 10.8 months vs. 7.5 months (HR, 0.61; 95% CI, 0.49–0.77; *p* < 0.001), and PFS, 5.6 vs. 2.4 months (HR, 0.44; 95% CI, 0.36–0.54; *p* < 0.001), even in the population that had already received bevacizumab in earlier treatment lines. Toxicity was significantly higher in the combination arm, but in both studies, RECOURSE and SUNLIGHT, the overall rate of adverse events was high, especially for hematological alterations (neutropenia, anemia), fatigue and diarrhea. It is important to note, however, when selecting patients for the use of FTD/TPI as monotherapy or in combination, that the population in the SUNLIGHT study is very different in terms of characteristics compared to the RECOURSE study, with the latter being heavily pretreated [12]. Moreover, current research suggests the use of the FTD/TPI combination in earlier treatment lines [13,14,15]. The optimization of the use of this molecule also includes understanding not only the best combination but also the subpopulation that could benefit from it. For example, a study presented at ESMO 2024, which combined FDT/TPI with Ramucirumab in a population with refractory mCRC, suggested that the potential benefit of such a combined therapy might apply to a specific category of patients. In the case of the aforementioned combination, this advantage was observed particularly in women and patients with left-sided colon cancer [16].

#### 2.1.3. FRESCO2 and Fruquintinib

Fruquintinib is a selective oral VEGFR1, -2 and -3 inhibitor approved by the FDA and EMA for the treatment of patients with refractory mCRC. The FRESCO-2 study is an international, multicenter, randomized, double-blind, placebo-controlled trial that evaluated the efficacy of fruquintinib in terms of OS and PFS. Patients received fruquintinib orally once a day for the first 21 days of a 28-day cycle, compared to placebo + BSC, in a heavily pretreated patient population [17]. The median OS was 7.4 months for the Fruquintinib + BSC group compared to 4.8 months with placebo + BSC (HR, 0.66; 95% CI, 0.55–0.80; *p* < 0.001) and PFS of 3.7 months for the Fruquintinib group compared to 1.8 months for placebo (HR, 0.32; 95% CI, 0.27–0.39; *p* < 0.001). Again, the percentage of patients with adverse events was high, especially fatigue, hand-foot syndrome and hypertension, leading to treatment discontinuation in 20% of patients, dose interruptions in 47% and dose reductions in 24% of cases [17]. It is interesting to note that, in this study, a very high percentage of patients (73%) had received more than three treatment lines in the metastatic setting, with 100% of patients having previously received regorafenib or FTD/TPI, and some of them both. This is an extremely pretreated population, so despite the advantage in terms of OS and PFS compared to placebo, and considering the toxicity, it is important to note that no hematological toxicity was reported, despite the potential hematological and bone marrow reserve impairment in the population.

Interesting studies on the combination of fruquintinib with FTD/TPI or similar drugs have been conducted in heavily pretreated populations, from the third line onwards. This combination has shown greater activity than single-agent therapies in preclinical models [18] and has been proposed in both prospective and retrospective clinical studies [19,20].

**Table 1:** Drugs currently indicated for refractory mCRC.

Study	Treatment	N pts	mOS	HR mOS	mPFS	HR mPFS	RR (%)	Main AEs	Prior Biologics
CORRECT Grothery et al. [6]	Regorafenib	505	6.4	HR 0.77 *p* = 0.0052	1.9	HR 0.49 *p* < 0.0001	1.0	HFSR Fatigue	100% bevacizumab 100% anti-EGFR
RECOURSE Mayer et al. [9]	TAS-102	534	7.1	HR 0.68 *p* < 0.0001	2.0	HR 0.48 *p* < 0.0001	1.6	Neutropenia Diarrhea	100% bevacizumab 100% anti-EGFR
FRESCO Li et al. [19]	Fruquintinib	278	9.3	HR 0.65 *p* < 0.001	3.7	HR 0.26 *p* < 0.001	4.7	HFSR HTN	30% bevacizumab 14% anti-EGFR
SUNLIGHT Prager et al. [11]	TAS-102 + Beva	246	10.8	HR 0.61 *p* < 0.001	5.6	HR 0.44 *p* < 0.001	6.3%	Neutropenia HTN	71% bevacizumab 94% anti-EGFR
FRESCO-2 Dasari et al. [17]	Fruquintinib	516	7.1	HR 0.66 *p* < 0.001	3.7	HR 0.32 *p* < 0.001	2%	HFSR HTN	97% anti-VEGF 39% anti-EGFR
RAMTAS Annals, 2024 [16]	TAS-102 + Ramucirumab	213	7.46	HR 0.871 *p* = 0.1941	2.37	HR 0.774 *p* = 0.0110	1.9%	Neutropenia Leukopenia	87% bevacizumab

### 2.2. Ongoing Clinical Trials with RAS Inhibitors

The viral oncogenic proteins of rat sarcoma (RAS) belong to the GTPase protein family and consist of four members encoded by three genes (KRAS4a, KRAS4b, HRAS and NRAS), which share sequence homology and differ from each other by the presence of the hypervariable C-terminal region. Oncogenic mutations in these oncoproteins lead to GTP hydrolysis, resulting in persistent binding that triggers the subsequent activation of downstream signaling pathways, such as Raf/MEK/ERK, which continuously stimulate survival. The KRAS mutation has always generated considerable interest regarding the potential to exploit its positivity, as it is one of the most important oncogenic mutations in colorectal cancer (CRC). Much scientific research has been invested in potential targeted drugs aimed at inhibiting its activity, thus significantly impacting the natural progression of these malignancies, which typically have a poor prognosis. Indeed, the presence of the KRAS mutation indicates a more aggressive disease with worse prognosis [21]. Until recently, however, KRAS was considered non-targetable because in vitro studies did not yield promising results. In recent years, scientific research has started to present the first encouraging data on the clinical activity of KRASG12C inhibitors, particularly adagrasib and sotorasib, in terms of objective response rate (ORR) in heavily pretreated KRAS G12C-mutated mCRC populations [22,23].

Despite the encouraging data from the aforementioned phase Ib/II studies, it became clear early on that the KRASG12C inhibitor treatment was less effective and impactful compared to what has been observed in other diseases, such as KRASG12C-mutated lung cancer [24]. This was attributed to the specific molecular characteristics of colorectal cancer and the complex signaling pathways, as demonstrated by preclinical studies [25]. Specifically, it was described that, in colorectal cancer cell lines, the inhibition of KRASG12C induces a greater rebound of phospho-ERK compared to NSCLC cells, with reactivation of EGFR. EGFR-mediated signaling was identified as the dominant mechanism of resistance to KRASG12C inhibitors in colorectal cancer. Therefore, a combination therapy targeting both EGFR and KRASG12C was proposed to overcome resistance to KRASG12C blockade in colorectal cancer [26]. Both monoclonal anti-EGFR antibodies used in current clinical practice for metastatic colorectal cancer treatment, Cetuximab and Panitumumab, have been studied in combination with KRASG12C inhibitors. Specifically, Cetuximab was studied in combination with Adagrasib in the KRYSTAL-1 study, a phase I/II study that included 94 patients with pretreated metastatic CRC with KRAS G12C mutation. These patients were administered Adagrasib 600 mg twice daily in combination with Cetuximab at an initial loading dose of 400 mg per square meter of body surface area, followed by 250 mg per square meter weekly or 500 mg per square meter every two weeks [27]. The results were encouraging, with an ORR of 34%, a median PFS of 6.9 months and a median OS of 15.9 months. Consistent with these findings, the combination of Panitumumab with a KRAS G12C inhibitor, particularly Sotorasib, showed positive results in phase Ib/II studies, which were later confirmed in the phase III CodeBreaK 300 study. This study included 53 patients with advanced CRC with KRAS G12C mutation refractory to chemotherapy [28]. In this study, Panitumumab was studied in combination with Sotorasib at the standard dose of 960 mg and in combination with a reduced dose of 240 mg, compared to a control arm receiving standard treatment for refractory mCRC (trifluridine-tipiracil or regorafenib). The best results were seen with the Panitumumab + Sotorasib combination at the standard dose of 960 mg, showing an ORR of 26.4% and a median PFS of 5.6 months, with a significant advantage over the reduced Sotorasib dose combination and, most notably, over the standard treatment arm, which showed an ORR of 0% and a median PFS of 2.2 months.

Numerous trials are ongoing investigating combinations of monoclonal anti-EGFR antibodies with other KRAS G12C inhibitors [29,30]. To date, the best molecule is not known as there are no comparative studies, given the similar toxicity. Despite the promising data from combination studies with KRAS inhibitors and anti-EGFR monoclonal antibodies for patients with refractory mCRC, many questions remain unanswered. In particular, it will be interesting to understand whether the optimal regimen of such combinations in KRAS G12C-mutated disease should be proposed in earlier treatment lines, and if so, whether it should be combined with chemotherapy and with which safety profile. Some combination trials with chemotherapeutic agents are ongoing [31,32,33]. Considering the complexity of the downstream EGFR signaling, many studies have explored the potential use of inhibitors targeting other oncoproteins involved in signaling mechanisms of proliferation and cell survival in patients with KRAS G12C mutations, such as SHP2, SOS and MEK. The data from these studies are promising and interesting but still immature [34,35,36].

Since KRAS G12C mutation is not the most frequent in colorectal cancers, unlike lung cancer, considerable interest is focused on other potential KRAS inhibitors, particularly KRAS G12D, which represents the most common KRAS mutation in colorectal cancer. Currently, there is encouraging preclinical data on ASP3082, a KRAS G12D degrader, and a phase I/II study (NCT05382559) is ongoing. Another KRAS G12D inhibitor under investigation is MRTX1133, in a phase I/II clinical trial (NCT05737706). Other KRAS inhibitors being studied are pan-inhibitors, which should have a broad spectrum of action. It will be interesting to monitor their development and safety data, as well as understand in which treatment lines these molecules will be placed [37,38] (see Table 2).

### 2.3. Ongoing Clinical Studies with Combination Therapies Using Immune Checkpoint Inhibitors

Immunotherapy is a well-established standard treatment for patients with MSI-H mCRC, both in the first-line setting and in subsequent lines if not yet used [39,40]. This latter situation is now rare in clinical practice as patients eligible for treatment with immune checkpoint inhibitors are typically treated early. However, interesting data have emerged in the setting of heavily pretreated patients with microsatellite stable disease. It is known that this molecular footprint renders patients resistant to immunotherapy, but since they represent the majority of colorectal cancer cases, numerous efforts have been made to increase the percentage of patients susceptible to immunotherapy by combining with immunomodulatory drugs to overcome this resistance. In particular, it is believed that drugs acting on the tumor microenvironment may favor antigen expression and immune cell recruitment. Combination therapies with chemotherapy regimens have been studied, yielding interesting data, especially in terms of better patient selection for the combination, such as a tool used in the Atezotribe study, which utilized a signature of 27 immune-related genes that could predict greater sensitivity to immunotherapy [41]. However, no chemo-immunotherapy combinations for MSS mCRC patients have been approved in current clinical practice due to the lack of statistically significant efficacy data [42]. A recent very interesting study conducted in the refractory, heavily pretreated disease, which was not amenable to targeted therapy, is the phase I study evaluating the combination of Botensilimab, a multifunctional anti-CTLA-4 antibody with Fc-enhanced activity, in combination with Balstilimab, an anti-PD-1 antibody [43]. The rationale behind this combination is the potential activity of Botensilimab, designed to expand therapy to poorly immunogenic solid tumors such as MSS mCRC. Several interesting data have emerged from this study, particularly regarding patients with liver metastases. The population in this study was heavily pretreated, with a median of three prior therapeutic lines. About 17% of the population had active liver metastases, with the remaining 83% having no history of liver metastasis, and 14% having had liver disease treated locally (surgery or other locoregional treatment). The data from this study show more favorable outcomes for patients with non-hepatic metastatic disease, with ORR (22% vs. 0%), DCR (73% vs. 25%), PFR (4.1 vs. 1.4) and OS (20.9 vs. 7.4 months) [43]. These data reinforce findings from other studies, both clinical and preclinical, on resistance of liver disease to immune checkpoint inhibitor treatments [44,45], suggesting a possible additional factor to consider in patient selection (see Table 2).

### 2.4. Ongoing Clinical Studies with Drugs Targeting Other Specific Mutations

#### 2.4.1. ERB2 Amplification

Good clinical practice encourages oncologists to enroll patients in clinical trials to provide access to targeted treatments. However, this is often challenging in heavily pretreated patients due to factors such as poor performance status (ECOG PS ≥ 2) and the focus of most trials on early treatment lines.

Several ongoing trials are exploring anti-HER2 therapies for mCRC patients with HER2 amplification, which occurs in about 5% of KRAS wild-type cases [46]. Studies in advanced treatment lines have shown promising response rates, supporting the role of targeted therapies. The HERACLES study demonstrated a 28% response rate with trastuzumab and lapatinib in HER2-overexpressing KRAS wild-type mCRC patients [47]. Similarly, the MyPathway study achieved a 32% response rate using trastuzumab and pertuzumab [48]. In contrast, the TAPUR study showed no benefit in patients with ERBB2/3 mutations but reported a 25% response rate in those with ERBB2 amplification [49].

Antibody-drug conjugates (ADCs), combining monoclonal antibodies with cytotoxic agents, have emerged as promising treatments, with agents like trastuzumab emtansine and trastuzumab deruxtecan showing potential [50,51]. These therapies are of particular interest due to the poor prognosis and reduced response to anti-EGFR drugs in RAS wild-type mCRC. Ongoing research aims to clarify the role and sequencing of anti-HER2 and anti-EGFR therapies in this subset of patients (see Table 2).

#### 2.4.2. Braf V600E

Patients with mCRC (metastatic colorectal cancer) with BRAF mutation represent about 12% of metastatic colorectal tumors and generally have a more unfavorable prognosis, mostly affecting the right colon, often with microsatellite instability [52]. Compared to melanoma, treatment with BRAF inhibitors has a modest response rate, which should again be interpreted in light of the complexity of the signaling pathways and the key players in tumor oncogenesis in colorectal cancers. This has led to the need for combined treatments with other drugs, particularly chemotherapy and anti-EGFR antibodies. The BEACON trial demonstrated the efficacy of combining BRAF, MEK and EGFR inhibitors, with Encorafenib plus Cetuximab now considered the gold standard due to similar overall survival (OS) to the triplet regimen but with a better safety profile [53]. However, response duration remains unsatisfactory, prompting research into novel combinations, such as immune checkpoint inhibitors, which may enhance tumor immunogenicity [54]. Ongoing studies are exploring combinations targeting other pathways, such as PI3K inhibitors, to overcome resistance and improve outcomes [55,56,57,58]. Studies are underway to evaluate the efficacy and safety profile of these combinations (see Table 2).

#### 2.4.3. NTRK

Kinase gene fusions, though rare (about 0.9% in colorectal cancers), are promising therapeutic targets, with NTRK being the most common, followed by RET, FGFR, ROS1 and ALK [59]. NTRK fusions are more frequent in MSI-high and RAS/BRAF wild-type tumors, showing clinical similarities to BRAF mutations, such as right-sided primary tumors and higher metastatic potential [60]. These fusions are associated with poor prognosis and resistance to EGFR inhibitors, increasing their scientific relevance.

The NTRK inhibitors larotrectinib and entrectinib have demonstrated efficacy in metastatic solid tumors, including colorectal cancer, with FDA approval based on data from phase I/II “basket” trials [61,62] (see Table 2).

However, resistance mechanisms have been identified: on-target (mutations reducing drug affinity) and off-target (activation of alternative pathways like BRAF and KRAS mutations). Second-generation inhibitors, such as selitrectinib and repotrectinib, have shown promise in overcoming on-target resistance, but off-target resistance remains a challenge, especially in colorectal cancer where MAPK signaling activation is common. Research is ongoing to develop targeted drug combinations to address these resistance mechanisms and improve treatment outcomes [63,64] (see Table 3).

**Table 2:** Targeted therapies for refractory mCRC.

Study	Interventions	Patients (n)	ORR (%)	mPFS (m)	mOS (m)	Grade ≥ 3 AEs (% of Patients)
HERACLES-A (phase II) [47]	Trastuzumab + lapatinib	35	30	4.7	10.0	22%, including fatigue (9%) and LVSD (6%)
MyPathway (phase II) [48]	Trastuzumab + pertuzumab	57	32	2.9	11.5	37%, including hypokalemia (5%) and abdominal pain (3%)
HERACLES-B (phase II) [51]	Pertuzumab + T-DM1	31	9.7	4.1	NR	Thrombocytopenia (6%)
TRIUMPH (phase II) [65]	Trastuzumab + pertuzumab	30	33 (ctDNA evaluable)	3.1	8.8	NR
TAPUR (phase II) [49]	Trastuzumab + pertuzumab	28	25; DCR 54	12 weeks	60 weeks	10.5%, including anemia, infusion reaction, diarrhea, LVSD, and lymphocyte count reduction
DESTINY-CRC01 (phase II) [50]	T-DXd 6.4 mg/kg	53	45.3	6.9	15.5	85%, including neutrophil count reduction (22%), anemia (14%), and platelet count decreased (10%)
DESTINY-CRC02 (phase II) [66]	T-DXd 6.4 mg/kg	40	27.5	5.8	NE	41%, including neutrophil count reduction (26%), anemia (27%), and platelet count decreased (10%)
	T-DXd 5.4 mg/kg	82	37.8	5.5	13.4	49%, including neutrophil count reduction (29%) and nausea (7%)
DESTINY-PanTumor01 (phase II) [67]	T-DXd 5.4 mg/kg	20	20	NR	NR	NR
MOUNTAINEER (phase II) [68]	Trastuzumab + tucatinib	84	38.1	8.1	23.9	Hypertension (7%) and urinary tract infection (6%)
	Tucatinib	30	-	NR	21.1	AST increase (6.7%)
HER2-FUSCC (phase II) [69]	Trastuzumab + pyrotinib	18	22.2	3.4	NR	Diarrhea (65%)
CodeBreaK 100 (phase II) [22]	Sotorasib	62	9.7	4.0	10.6	12%, including diarrhea, fatigue, increased alanine aminotransferase, and increased aspartate aminotransferase
CodeBreaK 300 (phase III) [28]	Sotorasib 960 mg + panitumumab	53	30.2	5.6 vs. 2.0	NE vs. 10.3	38.5%, including dermatitis acneiform (7.7%), hypomagnesemia (5.7%), and rash (7.7%)
	Sotorasib 240 mg + panitumumab	53	7.5	1.8 vs. 10.6	NE vs. 10.3	7.5%, including dermatitis acneiform (1.9%) and hypomagnesemia (1.9%)
KRYSTAL-1 (phase I–II) [27]	Adagrasib	44	19	5.6	19.8	27%, including diarrhea (9%) and fatigue (7%)
	Adagrasib + cetuximab	32	46	6.9	13.4	16%, including diarrhea, dermatitis acneiform, stomatitis, and infusion-related reactions
BEACON (phase III) [70]	Encorafenib + cetuximab + binimetinib	224	26.8	4.5 vs. 1.5	9.3 vs. 5.9	65.8%
	Encorafenib + cetuximab	220	19.5	4.3 vs. 1.5	9.3 vs. 5.9	57.4%
	Chemotherapy	221	1.8	—	—	64.2%
Yaeger et al. [71]	Vemurafenib + panitumumab	15	13.0	7.6	3.2	20%
Morris et al. [56]	Encorafenib + cetuximab + nivolumab	23	48.0	15.1	7.4	19%
Tabernero et al. [58]	Encorafenib + cetuximab + alpelisib vs. Encorafenib + cetuximab	52 vs. 50	27 vs. 22	15.2 vs. not reached	5.4 vs. 4.2	79% and 58%
SWOG S1406 [72]	Vemurafenib + cetuximab + irinotecan vs. cetuximab + irinotecan	50 vs. 56	17 vs. 4	9.6 vs. 5.9	4.2 vs. 2.0	30% vs 7%
Hong et al. [31]	Larotrectinib	153 (8 colon)	79 (50 colon)	25.8	44.4	13%
Demetri et al. [73]	Entrectinib	121 (10 colon)	61 (20 colon)	13.8 (2.8 colon)	33.8 (16 colon)	Increased weight, anemia, nervous system disorder

NR = not reported.

## 3. Patients with Heavily Pretreated mCRC Not Eligible to Target Therapy or Clinical Trials

These patients represent a significant portion of the most complex cases to manage in daily clinical practice, both in terms of therapeutic choice and management of toxicities. As we saw in the previous chapters, several molecules have been approved for the setting of advanced, refractory colon disease. However, the best order of use is still unknown, as these molecules have only been compared in retrospective studies [74]. What we do know is that the survival rates for colorectal cancer have significantly increased in recent years, and this has been linked not so much to improved first-line treatment but to the survival benefit gained from the sum of subsequent lines. This makes sequential therapies for colorectal cancer crucial, and it will be important in the near future to determine how to best propose therapies in treatment lines. The challenge will not only be to better select patients for targeted treatments based on a more thorough molecular understanding of the disease, thus enabling better treatment personalization, but also, with regard to innovative treatments, making thoughtful choices based on the therapies already received and the expected toxicities. Effective communication with the patient will become increasingly important in clinical practice, as patients need to be informed about potential options, and the choice should be shared. In this futuristic scenario, which points to increasingly complex molecules and treatment combinations, often with significant toxicities, it is crucial not to overlook what we have learned so far about this complex disease, including with respect to older chemotherapeutic treatments and rechallenging therapies already received. This includes the use of drugs such as mitomycin C, as well as rechallenging with Oxaliplatin and anti-EGFR drugs. Below is a brief summary of these potential scenarios.

### 3.1. Role of Treatments Rechallenge

The reintroduction of treatments previously received in earlier lines of therapy is not always feasible. It assumes, on one hand, that there was a response to the therapy, no progression during treatment and the absence of residual toxicities that would prevent reintroduction. This is the case, for example, with Oxaliplatin, a cornerstone drug in early chemotherapy lines, which is often suspended before progression, with treatment continuing with fluoropyrimidine, with or without a maintenance biologic drug, to reduce the likelihood of long-term neurological toxicity or significant hematological toxicities. Reintroduction in more advanced treatment lines is possible but constrained by residual peripheral sensory toxicity or limited bone marrow reserve, particularly thrombocytopenia.

Regarding Oxaliplatin, there is also the rechallenge strategy, which involves using the drug previously administered in earlier lines with an initial response to treatment, followed by progression that required a change in therapy. In the third or later line of chemotherapy, rechallenging allows for the reuse of Oxaliplatin, even after documented resistance, based on the assumption that, after one or more subsequent treatments, different from Oxaliplatin, the disease may have developed new mutations that make it responsive again to the drug. There are no prospective clinical studies in the literature, but retrospective real-life data seem to support this hypothesis [75,76,77]. Clinical trials are ongoing to evaluate the reintroduction/rechallenge of Oxaliplatin in patients with refractory mCRC, either in combination with biologic drugs, with fluoropyrimidine or as monotherapy (NCT03485027, NCT03940131, NCT03311750).

The possibility of rechallenging previously received treatments, despite documented progression, has always been of interest to oncologists, not only for necessity but also for the opportunity to use drugs already administered to the patient, with their toxicity already understood. In fact, we know that the molecules currently approved for refractory disease are often associated with high expected toxicity, and we do not always treat patients with a good ECOG PS. Often, marked asthenia or poor bone marrow reserve persist due to the accumulation of toxicities from prior treatments. We know that these molecules have been compared to placebo, but we lack objective data on the potential benefit compared to rechallenge treatments.

Some interesting data, although retrospective, seem to indicate that rechallenge therapy should not be considered inferior. One such retrospective study collected data from 394 patients across 21 centers, comparing PFS and OS in patients who, in the third line, received rechallenged fluoropyrimidine-based chemotherapy versus the third line with Regorafenib. It concluded that disease control rates and OS were higher with rechallenge therapy compared to Regorafenib, particularly in patients who had achieved disease control with fluoropyrimidine chemotherapy in one of the first two treatment lines [78].

One limitation in interpreting these data—aside from the retrospective nature of the study—is that the combination with biologic drugs (anti-EGFR or anti-VEGF) was allowed, with about 60% of patients receiving a biologic agent. This makes it difficult to discern whether the benefit of the rechallenge treatment was primarily due to the chemotherapy or the biologic treatments.

Several data now reinforce the idea that rechallenging biologic treatments has a rationale. Regarding anti-EGFR treatments, many retrospective studies had already suggested the potential efficacy of rechallenging therapy in subsequent lines for refractory mCRC [79,80,81], validated by prospective studies [82,83,84]. This potential benefit is based on molecular mechanisms that restore sensitivity to anti-EGFR treatment, particularly the restoration of Ras WT clones after a sufficient period from discontinuing anti-EGFR treatment. This is because the most frequent and well-studied mechanism of resistance to biologic treatment, particularly through tumor DNA (ctDNA) analysis via liquid biopsy, is the increase in KRAS-mutated disease. However, other resistant mutations may also occur, such as HER2 overexpression, ERBB2 amplification, BRAF V600E mutation and PI3K mutations. Therefore, it would be advisable to retest patients who were initially resistant to anti-EGFR treatment, following the mutation panel of refractory mCRC disease, to select patients who may benefit from such treatment [85] (see Table 4).

### 3.2. “Old Drugs”: The Case of Mitomycin C Combined with Capecitabine

Mitomycin C is an “old” oncological drug, a natural antibiotic administered intravenously that has shown antitumor activity and was used in a variety of solid tumors, including gastrointestinal cancers. It is usually a well-tolerated drug, although in some cases it can cause delayed bone marrow toxicity. The biological activity of mitomycin C in the treatment of colorectal cancer is well-known. In 2004, Rao and colleagues published a phase II study in a first-line metastatic colorectal cancer (mCRC) setting, demonstrating a benefit both in terms of overall response rate (ORR 38%) and progression-free survival (PFS 7.11 months), with a median overall survival (OS) of 14.3 months and no significant toxicity compared to capecitabine alone [93]. The authors argued that the benefit obtained with the combination of mitomycin C was supported by the data from the capecitabine monotherapy study, where the median OS was 12.9 months and the PFS was 4.6 months [94], concluding that this combination could be a valid therapeutic option in patients who prefer or cannot have a central venous catheter and are not candidates for other chemotherapy drugs. These data were replicated in an open-label phase II study that enrolled patients in third-line treatment (36 patients) who received the combination of mitomycin C and capecitabine. This study recorded a median overall survival of 9.3 months with a one-year survival rate of 30.6% and a median PFS of 5.4 months [95]. In clinical practice, several retrospective studies have been conducted, showing good tolerance to the treatment and disease control consistent with the data from advanced treatment lines [96,97]. Contrary to these data, another retrospective study by Ferrarotto in 2012, which analyzed data from 109 heavily pretreated patients who received mitomycin C and capecitabine, recorded a median survival of 4.5 months, similar to that of patients receiving best supportive care [98]. This may partly be explained by the idea that selecting patients who might benefit from the therapy is crucial, even—if not especially—in such advanced treatment settings, personalizing the treatment, when possible, especially in cases where patients can be enrolled in clinical trials with preserved performance status and targetable mutations.

## 4. Discussion

**Figure 1:**
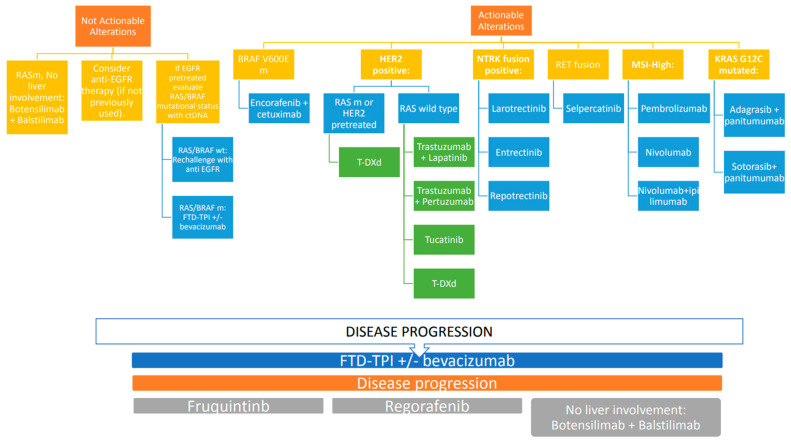
Therapeutic algorithm.

The treatment landscape for metastatic colorectal cancer (mCRC) continues to evolve, driven by the increasing complexity of disease biology, novel therapeutic options and the pressing need for personalized strategies. This review highlights key challenges in managing heavily pretreated mCRC patients, underscores the need for effective sequencing of existing therapies and explores emerging options, including rechallenge strategies and targeted combinations. Recent advancements in molecular profiling have reshaped the management of mCRC by enabling targeted therapies for specific genetic mutations, such as KRAS G12C, HER2 amplification and BRAF V600E. Despite promising data from clinical trials, the benefit of targeted therapies often remains limited in duration due to primary and acquired resistance mechanisms. For instance, KRAS G12C inhibitors like adagrasib and sotorasib have demonstrated efficacy in pretreated populations in combination with anti-EGFR agents [27,28]. Studies such as KRYSTAL-1 and CodeBreaK 300 highlight the potential of these combinations, yet further research is needed to optimize sequencing and define patient subgroups most likely to benefit. Rechallenge therapies represent an intriguing approach for managing refractory mCRC, always taking into account the residual toxicities from previous treatments and the expected toxicities. Retrospective studies and prospective trials suggest that rechallenging with anti-EGFR therapies can be effective in patients with restored RAS wild-type status, as determined by liquid biopsy [85]. Similarly, oxaliplatin rechallenge may offer benefit in select patients with preserved bone marrow reserve and manageable neurotoxicity, underscoring the importance of patient selection and molecular monitoring. However, the lack of large prospective studies comparing rechallenge with newer agents like regorafenib or trifluridine/tipiracil (FTD/TPI) limits definitive conclusions. Mitomycin C, combined with capecitabine, presents a potentially viable option in advanced treatment settings, particularly for patients ineligible for clinical trials or targeted therapies. Historical data demonstrate modest survival benefits and manageable toxicity profiles, yet conflicting results from retrospective studies highlight the need for careful patient selection. The observed variability in outcomes likely reflects differences in baseline patient characteristics and prior treatments, emphasizing the importance of shared decision-making and personalized approaches. Drugs like regorafenib, fruquintinib and FTD/TPI have shown survival benefits in refractory mCRC, as demonstrated in pivotal trials such as CORRECT, FRESCO-2 and RECOURSE [6,9,17]. All these molecules have demonstrated favorable quality-of-life outcomes compared to placebo in both clinical trials and retrospective real-life studies [99,100,101]. However, these agents are frequently associated with significant toxicities, including fatigue, hand-foot syndrome and hematological adverse events, which can limit their use in heavily pretreated patients. Strategies such as dose escalation for regorafenib (as validated by the ReDOS study) and combination therapies with bevacizumab (e.g., SUNLIGHT trial) are examples of how scientific research has focused in recent years on these two fundamental directions: increasing the effectiveness of treatments and reducing toxicities [8,11]. Further studies are warranted to refine these approaches and explore novel combinations, such as fruquintinib with FTD/TPI or immune checkpoint inhibitors [90]. Another aspect that has engaged research is aimed at increasing the number of patients eligible for immunotherapy treatments. In fact, while immune checkpoint inhibitors have revolutionized treatment for microsatellite instability-high (MSI-H) mCRC, MSS patients remain largely refractory to immunotherapy. Emerging combination strategies, such as Botensilimab and Balstilimab, aim to modulate the tumor microenvironment and enhance immunogenicity in MSS tumors [43]. Preliminary data from phase I studies indicate improved outcomes for patients without liver metastases, highlighting the need to better select patients in order to achieve an optimal response to immunotherapy treatment. The continued evolution of mCRC management hinges on the integration of novel therapeutic agents and combination regimens into clinical practice. Promising data from ongoing trials involving RAS inhibitors, HER2-targeted therapies and second-generation NTRK inhibitors underscore the potential for new targeted approaches [See Table 3]. Additionally, efforts to address off-target resistance mechanisms, optimize immunotherapy combinations and refine molecular profiling techniques will be crucial to improving outcomes [see Figure 1].

## 5. Conclusions

The management of heavily pretreated mCRC remains a complex challenge, requiring a nuanced understanding of disease biology, therapeutic options and patient-specific factors. While significant progress has been made with targeted therapies, immunotherapy and rechallenge strategies, optimizing sequential treatment regimens and minimizing toxicity are paramount. Comprehensive molecular profiling and collaborative decision-making between clinicians and patients are essential to advancing personalized care. As ongoing research continues to provide new insights, the ultimate goal remains to extend survival and enhance quality of life for this challenging patient population.

## Figures and Tables

**Table 3:** Ongoing clinical studies for refractory mCRC.

Trial	Intervention	Outcome	Safety (Grade 3–4)
Phase 1a–1b (Botensilimab + Balstilimab) NCT03860272.	Botensilimab (anti-CTLA4) + Balstilimab (anti-PD-1)	ORR: 17%, mPFS: 3.5 months, mOS: 20.9 months,	32%. Results more pronounced in patients without liver metastases
Phase 1b ACTIVATE-Colorectal (NCT05608044)	Botensilimab ± Balstilimab vs. Regorafenib or FTD–TPI	ORR: 22% 0%, mPFS 4.1 vs. 1.4, mOS 20.9 vs. 7.4 months	Data not provided
Phase 1b/2 OrigAMI-1 NCT05379595.	Amivantamab (EGFR–MET bispecific antibody) ± chemotherapy (FOLFOX or FOLFIRI)	Cohort A (L-sided, no anti-EGFR) (n = 17) mPFS 5.7 months, B (n = 54) (L-sided, post anti-EGFR) mpFS 3.75 months, C (n = 18)(R-sided, ±anti-EGFR) mPFS 3.5 months	The most frequent treatment-emergent adverse events were rash (84%) and infusion-related reactions (53%)
Phase 1 (Divarasib) NCT04449874	Divarasib (KRAS G12C inhibitor)	ORR: 29.1%, mPFS: 5.6 months.	11%. Grade 4 event: 1%. Dose reductions: 14%. Discontinuation: 3%
Phase 1b (Divarasib + Cetuximab) NCT04449874	Divarasib + Cetuximab	ORR: 62.5%, Median PFS: 8.1 months, Median duration of response: 6.9 months.	Dose reductions: 13.8%. No withdrawals due to AEs
Phase 1 NCT05382559	ASP3082 degrader selectively targeting KRAS G12D	Potentially active in KRASG12D-mutant CRC	AEs occurred in 69.4% pts, including 5% with grade 3. Among AEs were fatigue, infusion-related reactions, pruritus, nausea, urticaria, aminotransferases increased and vomiting
	RMC-9805 (covalent KRASG12D inhibitor)		
Phase 1/2 study NCT05737706	MRTX-1133 (non-covalent KRASG12D inhibitor)		
Phase 1–2 Combination therapies (BRAF/ICI) NCT03668431.	Encorafenib + EGFR inhibitors ± ICIs	ORR: 50%, mPFS: 7.4 months, mOS: 15.4 months.	18%
Phase 2 SWOG S2107 NCT02928224	Encorafenib + Cetuximab ± Nivolumab	Data not provided.	Data not provided
Phase 2 NCT03485027	Rechallenge chemotherapy (XELOX, FOLFOX, FOLFIRI, Irinotecan monotherapy)	ORR: 2.4%, mPFS 4.0 months (95% CI, 2.62–5.38). The data of OS was still immature.	11.76% neutropenia 9.7%, oxaliplatin related acute anaphylaxis 4.8%, diarrhea 4.8%
Phase 2 REPAN NCT03940131	KRAS/RAS wild mCRC after repeated RAS testing at last line of therapy, rechallenge panitumumab combined with chemotherapy similar to that given at 1st line (5-fluorouracil/leucoverin combined with oxaliplatin or irinotecan)	Data not available	Data not available

**Table 4:** Rechallenge with EGFR inhibitors.

Study	Interventions	Patients (n)	ORR (%)	mPFS (m)	mOS (m)	Grade ≥ 3 AEs (% of Patients)
Santini et al. [86]	FOLFIRI/irinotecan + cetuximab	39	53.8	6.6	NR	Skin rash (38.5%), neutropenia (18.0%) and diarrhea (7.7%)
JACCRO CC-08 [83]	Irinotecan + cetuximab	34	2.9	2.4 (3-month PFS 44.1%)	NR	Neutropenia (2%)
JACCRO CC-09 [84]	Irinotecan + panitumumab	25	8	3-month PFS 50%	NR	Acneiform rash (17%), hypomagnesemia (13%) and dry skin (13%)
CRICKET [82]	Irinotecan + cetuximab	28	21	3.4	9.8	Diarrhea (9%), skin toxicity (4%), neutropenia (4%) and HFS (7%)
E-Rechallenge [87]	Irinotecan + cetuximab	33	15.6	2.9	8.6	NR
CHRONOS [88]	Panitumumab	27	30	4.5	13.8	Skin rash (5%), folliculitis (6%)
PURSUIT [89]	Panitumumab + irinotecan	50	NR	3.6	NR	58.5%
CAVE Li [90]	Cetuximab + avelumab	32	6.5	3.6	11.6	Cutaneous eruption (14%) and diarrhea (4%)
VELO [91]	FTD-TPI vs. FTD-TPI + panitumumab	31 vs. 31	9.0 vs. 7.7	2.5 vs. 4.0, HR 0.48, 95% CI 0.28–0.82, *p* = 0.007	13 vs. 16, HR 0.54–1.17, *p* = 0.9	Rash (19%) in the experimental arm
CITRIC [92]	Irinotecan + cetuximab + investigator choice	27	12.9 vs. NR, *p* = 0.012	4.4 vs. 2.2, HR 0.72, 95% CI 0.40–1.28, *p* = 0.26	NR	Diarrhea (16.1%), skin rash (9.7%), mucositis (6.4%) and neutropenia (6.4%) in the experimental arm

NR = not reported.

## Data Availability

Data sharing not applicable.
